# Behavioral patterns in elderly single-person households

**DOI:** 10.1016/j.heliyon.2024.e39069

**Published:** 2024-10-10

**Authors:** David Araya, Carla Taramasco, Miguel Piñeiro, Anthony Fleury

**Affiliations:** a ITISB (Instituto de Tecnología e Innovación para la Salud y Bienestar), Facultad de Ingeniería, Universidad Andrés Bello, Viña del Mar, 2520000, Chile; b Núcleo Milenio de Sociomedicina, Santiago, 8320000, Chile; c IMT Nord Europe, Institut Mines-Télécom, Univ. Lille, Centre for Digital Systems (CERI SN), Lille, 59160, France

**Keywords:** Ambient Assisted Living (AAL), Behavioral pattern identification, Elderly activity monitoring

## Abstract

The global rise in the aging population and the increase in older adults living alone have raised concerns about health-related behaviors, particularly sedentary lifestyles and reduced daily activities. These behaviors are linked to higher risks of physical and cognitive conditions. While many global studies have explored these patterns, research within the Chilean context remains limited. This work presents a analysis of behavioral patterns in elderly individuals living alone in Chile, offering valuable insights into this population. Using clustering techniques, we identified two distinct activity patterns among the participants. The first pattern is characterized by a gradual increase in activity during the day, peaking around midday and followed by a decline, likely associated with meal preparation and rest. The second pattern demonstrates a more dynamic lifestyle, with a rapid surge in activity after waking and sustained levels throughout the day, suggesting a potentially healthier approach to aging. These findings align with previous studies indicating high levels of sedentary behavior in older adults, reinforcing the need for interventions tailored to diversify daily routines and promote physical activity. This study is the first to explore these patterns in the Chilean context, contributing to a more comprehensive understanding of elderly care and informing future strategies for improving the well-being of older adults living alone.

## Highlights

• Presents a novel analysis of behavioural patterns in elderly individuals who live alone in Chile.
• Identifies two distinct activity clusters among elderly living alone, showcasing varied lifestyle patterns through clustering techniques.
• Reveals significant sedentary patterns among elderly, urging the development of interventions to promote more active lifestyles.

## Graphical abstract

**Figure:**
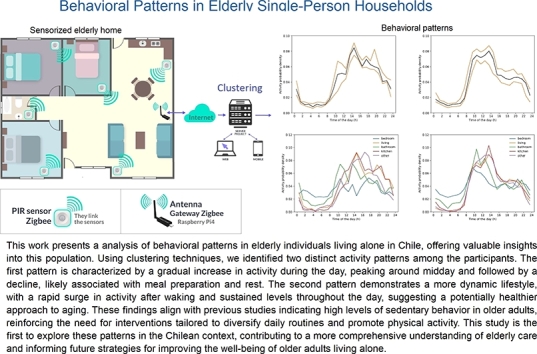

## 1 Introduction

The global population is aging at an unprecedented rate. The World Health Organization (WHO) reported that in 2023, the average global life expectancy reached 73.16 years, with figures exceeding 83 years in some developed countries [1]. According to data, about 17.34% and 21.21% of the population in the United States and European Union respectively, are 65 years or older in 2022 [2]. At the same time, there has been a significant worldwide increase in single-person households, accounting for 27.7% of the population in the United States, with 40.2% of said households being led by a person older than 65, as of 2020 [3]. Chile is experiencing similar demographic changes within this global trend. At present, 18% of the country's population is 60 years of age or older [4], projecting that in three decades the older people group will reach 31%, making Chile the second most aged country in Latin America [5]. On the other hand, the rate of single-person households in Chile is high, estimated at 18.1% [6].

The natural aging process entails a series of physical and cognitive problems that impact the functional capacity and autonomy of older adults. As physical and mental health deteriorates, chronic and neurodegenerative diseases, such as Alzheimer's and other dementias, begin to limit motor and cognitive abilities [7], [8]. These conditions make it difficult to carry out daily activities such as cooking, using the phone, or managing transportation, affecting their ability to live independently. The changes in daily routines caused by aging are initially subtle and difficult to perceive, both for the individuals themselves and for other family members. However, monitoring these patterns offers an opportunity to detect early anomalies before they become more serious issues. Ambient Assisted Living (AAL) technologies have emerged as a solution in this context, providing a range of functions from assisting users with physical or sensory disabilities to the intelligent management of home devices [9]. For example, in cases of neurodegenerative diseases such as Alzheimer's, it is crucial to identify early changes in daily behaviors, such as reduced physical activity, increased sleep fragmentation, or nocturnal agitation [9], [10], [11], [12]. Detecting these patterns can provide timely intervention, improving the quality of life for those affected and slowing the progression of the disease [13].

A key risk factor associated with health deterioration in older adults is the reduction in daily activities, resulting in sedentary behavior, the early detection of which can be crucial for preventing both physical and cognitive decline. Identifying these sedentary behaviors not only allows for monitoring their progression but also serves as an early indicator of physical and mental deterioration. Studies conducted in Japan have highlighted the need to monitor sedentary behavior in older adults, as this type of activity represents a considerable portion of their daily time [14]. On the other hand, a study in Portugal emphasized that, in addition to physical health, social capital and participation in social and cultural activities are essential for healthy aging [15]. Support networks and social interaction play a key role in improving the quality of life of older adults, showing that combating sedentary behavior is not only a matter of physical activity but also of social integration.

Ultimately, identifying and recording the daily activities of older adults is essential for developing more personalized intervention strategies. The literature widely supports the relationship between greater participation in daily activities and improved health perception [16]. Promoting physical, intellectual, and social activities are crucial for fostering healthy aging and preventing the decline associated with prolonged inactivity. However, the daily activities of older adults vary considerably according to cultural, social, and geographic contexts. Behaviors that are common in one country may not always be replicated in others, as factors such as customs, the physical environment, and family structures play a critical role in the everyday life of older adults. For this reason, it is not feasible to extrapolate these patterns from one context to another without local studies. In Chile, the lack of studies recording the behavioral patterns of older adults living alone limits the development of effective strategies. Therefore, this article contributes to a better understanding of the behavioral patterns of older adults in Chile, providing information for future research and potential interventions.

## 2 Related work and contribution

Monitoring the daily activities of older adults is important for identifying health patterns and potential risks associated with aging. Ambient Assisted Living (AAL) is a technology that combines sensors and algorithms to improve support for daily activities, particularly for the needs of the elderly [17], [18]. Sensors can collect data from the environment, including light, sound, temperature, pressure, and more [18], [10], as well as directly from individuals through wearables such as accelerometers, gyroscopes, magnetometers, and the like [19], [20], [21], [22].

Several significant studies have explored the use of Ambient Assisted Living (AAL) technology for monitoring and recognizing behavioral patterns in the elderly.

• A Brazilian study developed an unsupervised computational model using passive infrared sensors and a ZigBee network to monitor elderly individuals living alone, effectively identifying risk situations while preserving privacy with minimal computational cost [18]
• A study in the Emilia-Romagna region in Italy, employed PIR, pressure and Magnetic contact sensor where installed in the homes of post stroke patients. Their activities registered, using rule based preprocessing and through unsupervised algorithms their patterns compared to those reported in behavioral science as a quality of life indicator [10]
• the UbiCare system monitors environmental parameters and infers daily activities, although it does not focus on pattern recognition using real data [23]
• Another approach utilizes non-intrusive load monitoring (NILM) with smart meter data to monitor daily activities in the elderly, emphasizing cost-effective and less intrusive methods [24]
• research from the Washington State University Smart Home Project analyzes long-term behavioral patterns, employing anomaly detection and a fuzzy logic-based decision support system to alert caregivers [25]

The behaviors of older people living alone are strongly influenced by local cultural, social and environmental factors, because of this, previous work demonstrate the significance of the context where the data is obtained, [26], [27], [15], [14]. It is therefore crucial to recognize and understand these patterns in the specific context of each local reality. In Chile, for example, there is a lack of similar in-depth studies on these behaviors in the elderly population. Therefore, the value of this work lies in its focus on identifying these patrons within the Chilean context. This study to identify and characterize specific behavioral patterns of elderly individuals living alone in Chile, addressing the diversity and distinct profiles within this demographic, recognizing these unique aspects not only enriches the global understanding of elderly care, but also provide valuable insights for developing more effective and culturally appropriate care strategies.

## 3 Materials and methods

This section outlines the methodology for data acquisition, detailing the home setup, the type of data collected, and the storage process. It also describes data pre-processing procedures and analytical assumptions. The section concludes with general statistical information about the dataset and participants.

### 3.1 System architecture

**Figure 1:**
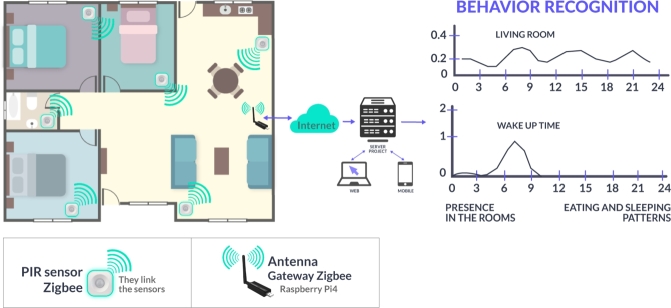
Schematic representation of the behavior recognition system. It illustrates the PIR sensors distributed in the rooms, the connection to the server, and the data analysis for identifying activity patterns.

The experiments were conducted in homes located in the Valparaíso region, specifically in the cities of Valparaíso and Viña del Mar. To monitor the daily activities of the participants, non-invasive passive infrared (PIR) sensors were employed in five rooms of the participants' homes, four sensors were in fixed rooms, beign the bathroom, kitchen, bedroom, living room and the fifth sensor was used, most commonly in a second bedroom o bathroom, depending on the layout of the house. A schematic layout of the sensor placement and system architecture is shown in Fig. 1. These sensors were integrated through a Raspberry Pi with HomeAssistant OS as remote station using the ZigBee protocol [28]. PIR sensors transmit their data over the the Raspberry Pi, who then sends the data to a central server. The information from each participant is subsequently saved in a database.

### 3.2 Data acquired

#### 3.2.1 Participants

The study was submitted and approved by the committee of bioethics of the university (Universidad Andres Bello). The participating individuals had to sign an informed consent form in compliance with the rules established by the bioethics Committee. Participants were interviewed and were required to complete four questionnaires, the first three are standardized questionaries for assesing the participants and the last one correspond to a questionnaire to check sociodemographic background:

• Barthel is a questianary to evaluate the ability to function independently and the mobility for day to day activities [29]
• EuroQol-5D (EQ-5D) is a standardized instrument for measuring general health status, often used in economic evaluations of healthcare [30]
• Sociodemograph pattern and clinical history

The results would be used to mesure if the participants meet the inclusion criteria, which are beign 60 year or orlder, to live alone, they didn't exhibit significant health issues like mobility limitations or severe cognitive decline and without pets.

#### 3.2.2 Global indicators on the dataset

The study population that met the inclusion criteria consists of individuals with varying levels of education, including those who have completed higher education and those who have not. Additionally, the participants represent diverse socioeconomic backgrounds, ranging from individuals with higher economic status to those from lower-income households.

Table 1 presents the different attributes of the participants. The dataset has 15 women and 3 men.

**Table 1:** Information on the elderly persons of the study.

Age	Gender	# of days
61	F	62
66	M	61
69	F	47
69	F	56
70	F	47
70	F	53
71	F	44
72	F	53
72	F	71
73	F	57
74	F	83
75	F	47
75	F	47
75	M	49
80	F	40
81	F	41
83	F	69
89	M	54

The mean age of participants was 73.6 years with a standard deviation of 6.3 years (73.0 ± 5.3 for female participants and 76.7 ± 9.5 for male participants).

### 3.3 Preprocessing of the data and assumptions

PIR sensors function by detecting changes in the infrared (IR) radiation emitted by objects within their field of view. One limitation of these sensors is that they are only capable of detecting movement activity and not the presence of the participants. This limitation prevents the sensors from detecting a person's presence when it's not moving, for instance, when sitting still on a chair.

To precisely determine the room in which participants were located, we preprocessed the data to address gaps that arose when participants remained stationary in a room. We filled these gaps based on the assumption that if a PIR sensor recorded activity, followed by a period of inactivity, and then activity resumed, without any other PIR sensor or entrance door sensor indicating activity during that time, the individual remained in the same room.

### 3.4 Identification of behavioral patterns

An effective way to identify behavioral patterns in large volumes of data is through clustering tools, which classify samples with similar characteristics. Previous studies have employed techniques such as hierarchical clustering, specifically HDBSCAN, to manage variable densities in data and generate clusters of different sizes and shapes [10].

In our study, we used the K-means method [31] to identify daily activity patterns in older adults. We chose this approach due to its simplicity and effectiveness in grouping data into categories that reflect general behaviors. To determine the optimal number of clusters, we used the silhouette method [32], which evaluates the cohesion and separation of the formed clusters, resulting in the selection of two groups as the best representation of activity patterns in the studied population.

## 4 Results of the analysis

### 4.1 Activities of the persons during the day

The daily activity level is a metric that can provide insights into a person's routines. By examining this metric over multiple days, one can uncover changes in their behavior over a broader timeframe.

**Figure 2:**
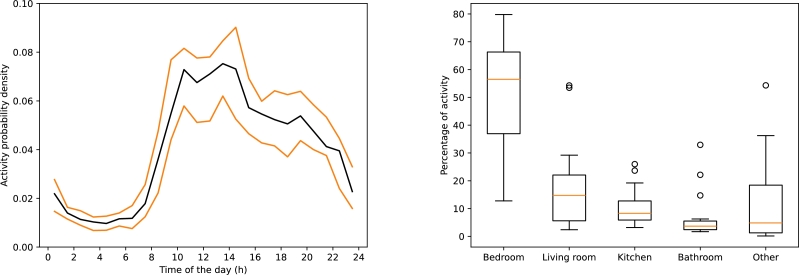
Activities of the persons depending on the time of the day (black line is the mean, orange lines are first and third quartiles (left) and Use of the rooms for the different persons of the dataset (right).

In Fig. 2, the overall activity level for the dataset is illustrated. This was calculated by initially determining the average activity level for every hour for each participant and subsequently averaging those individual hourly averages. A consistent trend is revealed from the analysis: activities typically commence at 9:00, peak around 12:00, and gradually decrease, reaching a minimum at around 23:00. The accompanying boxplot indicates that, on average, more than half of an individual's day is spent in the bedroom.

**Figure 3:**
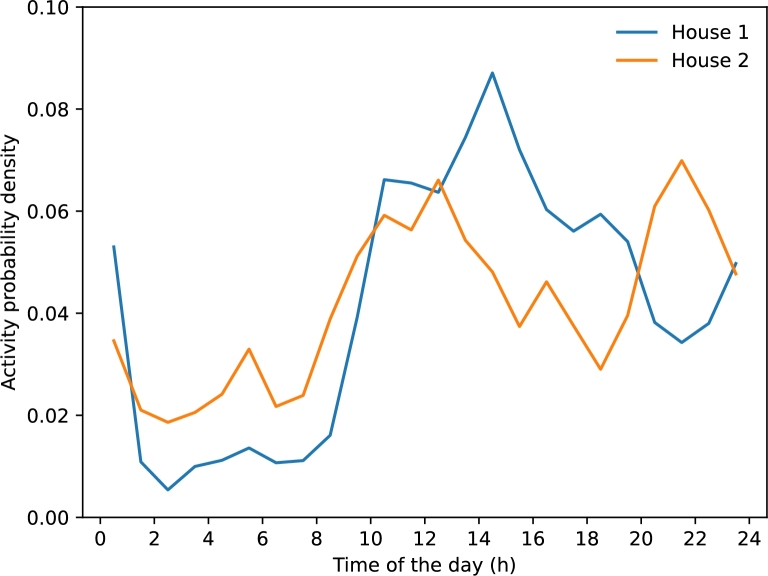
Comparative Daily Activity Curves of two Houses (subjects).

However, looking at overall activity levels can miss some differences. Fig. 3 shows the daily activities of two participants side by side. Their activities in the afternoon and before bedtime are quite different. This highlights that people in our study have varied habits. To see if everyone's habits were really different, we clustered the daily activity using the K-means method [31]. To determine how many clusters to build we used the silhouette method [32], which gave the highest score at 2 clusters, one with 5 participants and the other with 13.

**Figure 4:**
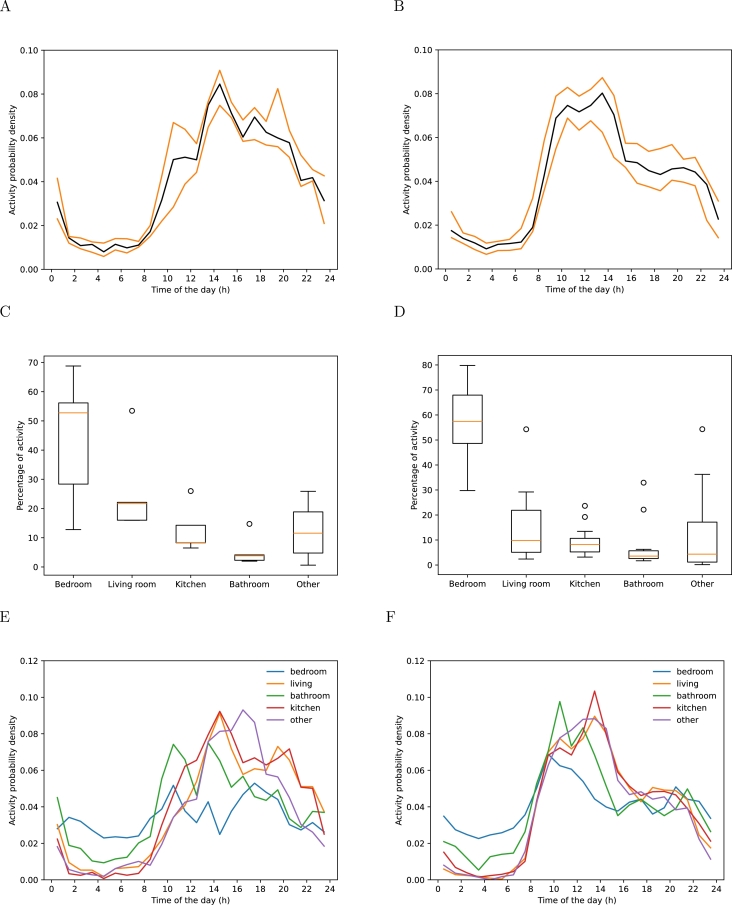
Level of activity for each hour for the first cluster (left) and second cluster (right) of persons.

Fig. 4 displays two distinct activity patterns in the left and right plots. Plot A shows a gradual increase in activity from wakeup time, peaking at 14:30, and then declining until 23:00. There is no significant increase in activity during the afternoon. In contrast, Plot B exhibits a rapid surge in activity from wakeup time, reaching its peak at 9:30 and maintaining a steady level until 14:00. Subsequently, it declines rapidly until 15:30, stabilizes until 21:30, and then steadily decreases until bedtime.

### 4.2 Occupancy of the different rooms

In Plots C and D, we evaluated the occupancy rates for various rooms within both clusters. We categorized the rooms into five types: the bedroom, living room, bathroom, and an “other” room, which could denote a second bedroom, dressing room, balcony, etc. In both clusters, the bedroom was the most frequently occupied room, followed by the living room, and then either the kitchen or the “other” room. The bathroom saw the least activity. Despite similarities in their patterns, the first cluster spends comparatively less time in the bedroom and more in the living room than the second cluster, indicating distinct lifestyle preferences between the two groups.

Subsequently, we plotted the usage patterns of different rooms throughout the day for each cluster. The first cluster exhibited a stronger time-dependent room preference, whereas the second cluster displayed less time-dependent room preference.

## 5 Wake-up time of the person

Inferring with the data that we have, we can try to determine at what time the person wakes up every day. For that, we assume that this time corresponds to the first firing of a sensor outside the bedroom (firings in the bedroom could be done during sleep time) and after 6 am. We have to note also that, as we can know from our knowledge of elderly persons, the wake-up time does not depend on the type of days. We tried to compute the same figure on weekend days versus weekdays, and the means and standard deviations are not significantly different.

This figure shows different subtleties of the data:

• Most of the subjects wake up relatively early (mean time before 7 am).
• The ones that have a mean value close to 6 and very little quantile values (17 and 18 for the more significant, but also 5, 6, and 11) may even wake up earlier or have non-usual behaviors at home during the night. We decided to start the detection at 6 am and if someone wakes up earlier, goes to another room, comes back in the room we will wait for another detection in another room to declare the wake-up. If there are activities in the bedroom it does not count. As a consequence, for some of the people, we have to find different behavior analysis algorithms, as it is not that simple to detect real wake-up from nycturia for instance (it could be a problem for other IoT devices such as watches that did not well resolve this issue).

**Figure 5:**
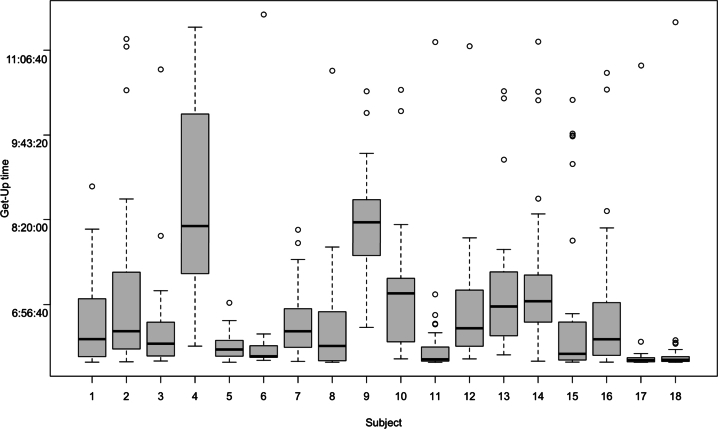
Wake-up time.

We can note also that, on another hand, we tried also to do the same analysis for the time at which the person goes to bed. Considering a period from 9 pm and searching for a one-hour period without another detection in another room than the bedroom, we did not have results that were close to the analysis done in Fig. 4 and consistent with it (Fig. 5).

## 6 Discussion

This study identifies the behavioral patterns in the daily routines of elderly individuals in Chile. The study observes key moments such as bedtime, which is typically around 23:00, and wake-up time, which is around 8 am. Additionally, two predominant behavioral patterns have been identified. The first pattern is characterized by a slower and more gradual increase in activity from wake-up time, culminating in a peak sustained for about an hour at 14:30, probably associated with lunch, followed by a decrease in activity. In contrast, the second pattern presents a rapid surge in activity from wake-up time, reaching its peak at 9:30 and maintaining a steady level until 14:00. This pattern likely corresponds to a diverse range of activities lasting for more than four hours, suggesting a more dynamic lifestyle and potentially a healthier approach to aging. These patterns not only reflect diverse daily routines but also indicate variations in restfulness and activity levels throughout the day among the elderly population in Chile.

Our findings are consistent with previous studies showing high levels of sedentary behavior in older adults [21]. In our analysis, we observed that a large portion of the participants' daily time is spent on activities such as meal preparation and resting, reflecting patterns similar to those found in the literature. For instance, studies conducted in Japan found that older adults spend a considerable part of their day in prolonged sedentary activities, such as watching television [14]. This suggests that sedentary behavior is a common pattern in this population, regardless of geographical or cultural context, reinforcing the need to develop strategies to promote physical activity and improve well-being in old age.

Similarly, our results align with research indicating that daily activity patterns in older adults tend to concentrate on a limited number of activities, mainly those related to the home [20]. In our study, we found that most of the older adults' time is spent in the kitchen, bedroom, and living room, highlighting the importance of monitoring these key spaces to identify possible changes in behavior. The predominance of these types of activities suggests that interventions to promote physical activity should focus on diversifying daily routines and incorporating movement into different contexts within the home.

However, while our results primarily focus on daily routines within the home, studies in Portugal have noted that, in addition to physical health, social capital and participation in social and cultural activities are essential for healthy aging [15]. This implies that any intervention aimed at reducing sedentary behavior should also consider the importance of social interaction and activities outside the domestic environment. Therefore, although our findings provide a relevant perspective on home-based activities, future research should incorporate these other factors to achieve a more comprehensive approach.

The differences in the identified activity patterns could be related to the health and well-being of older adults. The second pattern, characterized by more constant and prolonged activity throughout the day, could indicate a more active lifestyle, associated with better quality of life and healthy aging. This aligns with previous research that has highlighted the positive relationship between regular physical activity and better health perception [16]. The presence of this pattern suggests that maintaining a more dynamic level of activity could help prevent physical and cognitive decline. In contrast, the first pattern, which shows an earlier decrease in activity, could be linked to more sedentary habits, highlighting the importance of interventions aimed at promoting diversity and continuity in the daily activities of older adults.

One limitation of this study is the use of Passive Infrared (PIR) sensors, which, while effective for detecting movement within specific areas of the home, lack the ability to capture more nuanced activities such as resistance training or fine motor skills. Additionally, the sensors cannot distinguish between multiple individuals, making it difficult to accurately identify who is performing the activities. Identifying the number of people in the house is crucial as it simplifies information extraction and reduces the model's complexity. Another potential limitation is the presence of large pets, which could trigger false activations of the sensors, leading to data inaccuracies. Addressing these limitations could enhance the reliability of future monitoring systems.

The sample size and monitoring duration also pose potential limitations. Although our study provides an initial snapshot of daily behavioral patterns in older adults living alone, a more extensive sample and longer monitoring period would allow for a deeper understanding of how activity patterns evolve over time. We propose conducting a longitudinal study to observe the changes in rest and activity patterns in older adults over extended periods. This approach would help identify significant shifts in the fragmentation of rest and activity, providing a solid foundation for designing personalized interventions to improve the well-being of this population.

Moreover, incorporating additional contextual factors such as weather conditions, social interactions, and daily routines could offer a more comprehensive understanding of the behavioral patterns observed. Previous studies have highlighted the importance of integrating social and environmental contexts in monitoring systems to enhance the accuracy of predictions and interventions. Including these variables in future research could lead to more tailored strategies for promoting physical and cognitive health in older adults.

In terms of practical applications, the identification of activity patterns could be leveraged to develop real-time monitoring systems and early alert mechanisms for caregivers and healthcare professionals. This would enable prompt responses to potential health risks, such as sudden changes in sleep patterns or decreases in physical activity. Ultimately, this research lays the groundwork for future studies, aiming to refine these systems and incorporate additional factors to create a holistic approach to elderly care.

## CRediT authorship contribution statement

**David Araya:** Writing – original draft, Supervision, Methodology, Conceptualization. **Carla Taramasco:** Validation, Supervision, Conceptualization. **Miguel Piñeiro:** Writing – review & editing, Writing – original draft, Methodology, Formal analysis, Conceptualization. **Anthony Fleury:** Writing – review & editing, Writing – original draft, Supervision, Formal analysis, Conceptualization.

## Declaration of Competing Interest

The authors declare that they have no known competing financial interests or personal relationships that could have appeared to influence the work reported in this paper.

## Data Availability

The dataset used for this study is openly available at the following link: https://github.com/daraya78/Elderly_Behavior_Data_Chile. This repository provides the data files utilized in the research, allowing for transparency and further analysis.

## Appendix A Supplementary material

MMC 1 Processed Data from Motion Detectors includes time measurements for movements detected in respective rooms. MMC 1

MMC 2 Experimental Protocols detail the methods for data collection. MMC 2
